# Supplementary material for: Association between oral microbiome and seven types of cancers in East Asian population: a two-sample Mendelian randomization analysis
Source: Front Mol Biosci. 2023 Nov 21;10:1327893. doi: 10.3389/fmolb.2023.1327893 (PMC10702768; doi:10.3389/fmolb.2023.1327893)
Supplement: Supplementary file 1 [file DataSheet1.ZIP › Supplementary Materials/MR plots for tongue/tongue═╝/Lung cancer/pheno.1614_to_lung cancer_leave_one_out.pdf]

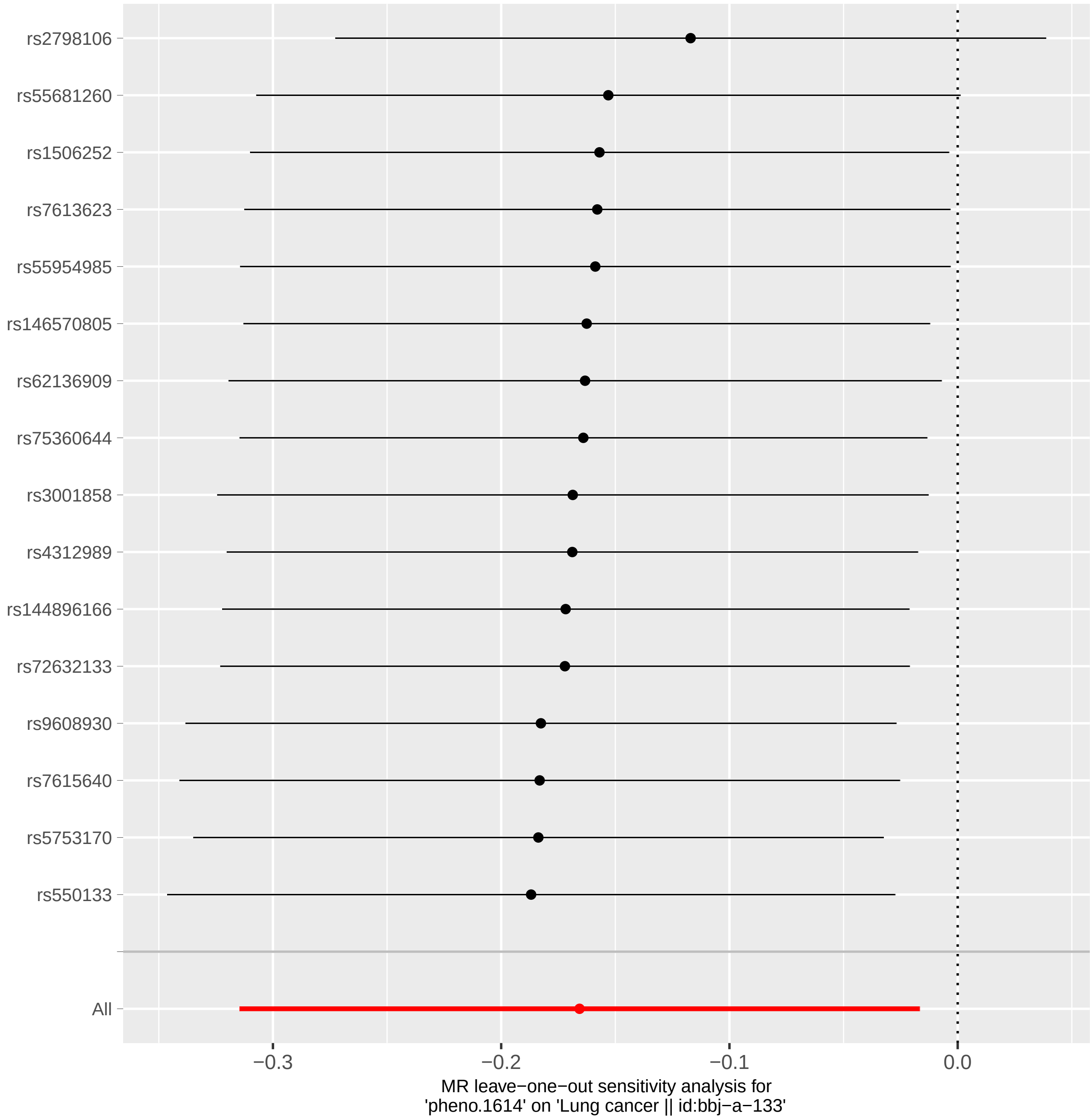
rs2798106
rs55681260
rs1506252
rs7613623
rs55954985
rs146570805
rs62136909
rs75360644
rs3001858
rs4312989
rs144896166
rs72632133
rs9608930
rs7615640
rs5753170
rs550133
All
−0.3
−0.2
−0.1
0.0
MR leave−one−out sensitivity analysis for
'pheno.1614' on 'Lung cancer || id:bbj−a−133'
